# Impact of left ventricular ejection fraction on clinical outcomes following ventricular tachycardia ablation: a propensity-matched analysis from a large multicentre database

**DOI:** 10.1093/ehjopen/oeag057

**Published:** 2026-04-09

**Authors:** Dharmindra Dulal, Ahmed Maraey, Paul Chacko, Tarvinder Dhanjal, Abhishek Maan, E Kevin Heist

**Affiliations:** Ohio State University Wexner Medical Center, Department of Internal Medicine, Columbus, OH 43210, USA; Division of Cardiovascular Medicine, University of Toledo Medical Center, Toledo, OH 43614, USA; Division of Cardiovascular Medicine, University of Toledo Medical Center, Toledo, OH 43614, USA; University of Warwick, Coventry, CV47AL, UK; Division of Cardiovascular Medicine, University of Toledo Medical Center, Toledo, OH 43614, USA; Cardiac Arrhythmia Service, Massachusetts General Hospital, Boston, MA 02114, USA

**Keywords:** Ventricular tachycardia, Catheter ablation, Left ventricular ejection fraction, Mortality, Outcomes

## Abstract

**Aims:**

Ventricular tachycardia (VT) ablation is an established therapy for patients with structural heart disease and recurrent VT. However, the impact of left ventricular function on peri-procedural and long-term outcomes remains incompletely understood. We evaluated the association of left ventricular ejection fraction (LVEF) on clinical outcomes after VT ablation.

**Methods and results:**

We conducted a retrospective cohort study using the TriNetX Research Network (2010–21) to evaluate outcomes after VT ablation, stratifying patients by LVEF (>30 vs. ≤30%). Propensity score matching (1:1) was used to balance baseline characteristics. The primary outcome was a 30-day composite safety endpoint defined as all-cause mortality, acute kidney injury (AKI), mechanical circulatory support (MCS) use, or cardiac tamponade. Secondary outcomes included 3-year all-cause mortality, ventricular arrhythmia recurrence, and rehospitalization. The individual components of the 30-day composite were evaluated in exploratory analyses. Among 2549 patients who underwent VT ablation, 623 were matched in each subgroup. The 30-day composite safety endpoint was significantly lower in patients with LVEF >30% (17.9 vs. 26.3%; *P* = 0.0004). In exploratory analyses, patients with LVEF ≤30% had higher 30-day mortality, AKI, and MCS use, while tamponade rates were similar between groups. At 3-year follow-up, all-cause mortality (15.2 vs. 28.7%) and rehospitalization (31.6 vs. 44.1%) remained significantly lower (*P* < 0.01) in the higher LVEF group. Ventricular tachycardia recurrence rates were high in both groups (71 vs. 67%) without a significant difference.

**Conclusion:**

In this large real-world study, patients with LVEF >30% undergoing VT ablation experienced significantly better peri-procedural and long-term outcomes.

## Graphical Abstract

**Graphical Abstract:**
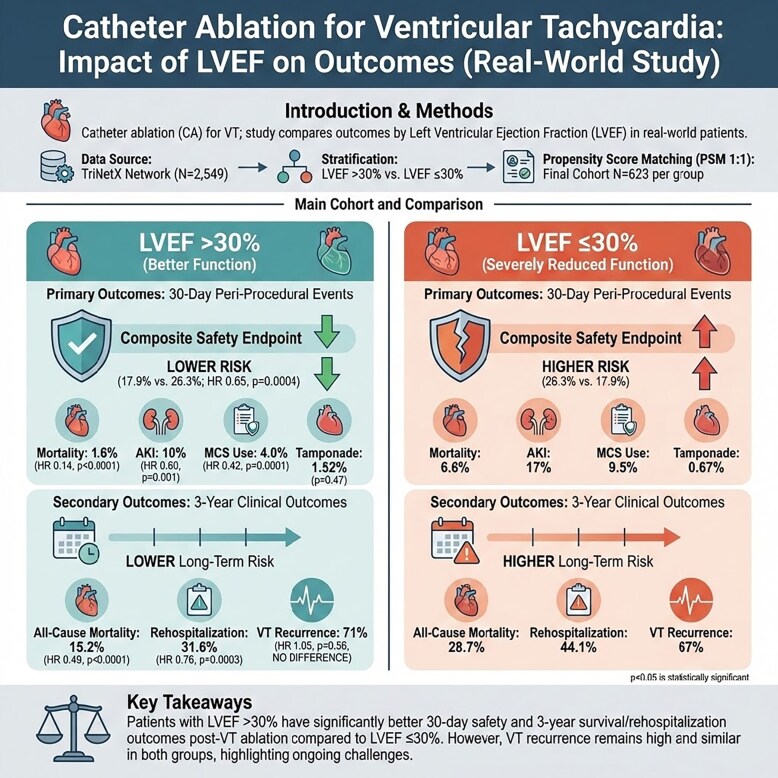

## Introduction

Catheter ablation (CA) has evolved to be a therapeutic option for ventricular tachycardia (VT), particularly in patients with recurrent arrhythmias or implantable cardioverter defibrillator (ICD) shocks.^1,2^ Several randomized trials have shown that CA can reduce the recurrence of VT and also that of ICD therapies, which could improve survival in selected high-risk populations.^3–6^ However, patients with severely reduced left ventricular ejection fraction (LVEF) were underrepresented in these randomized trials. The landmark trials such as SMASH-VT^3^ (The Substrate Mapping and Ablation in Sinus Rhythm to Halt Ventricular Tachycardia), VTACH^4^ (the Ventricular Tachycardia Ablation in Coronary Heart Disease), and VANISH^5^ (Ventricular Tachycardia Ablation versus Escalation of Antiarrhythmic Drugs) included patients with mild to moderately reduced LVEF, with limited representation of those with LVEF ≤30%. In contrast, the BERLIN VT^6^ (Preventive or Deferred Ablation of Ventricular Tachycardia in Patients with Ischemic Cardiomyopathy and Implantable Defibrillator VT) trial excluded patients with LVEF ≤30%. Therefore, there are limitations regarding applicability of trial results to the patients with advanced heart failure.

Patients with severely impaired LVEF (≤30%) also pose a particular set of challenges during VT ablation; as they often tend to have extensive arrhythmogenic substrate and experience a higher risk of peri-procedural complications including acute haemodynamic decompensation both during and after the ablation procedure.^7^ While VT ablation can reduce ICD shocks and overall VT burden, its impact on mortality in patients with severely reduced LVEF remains debatable. This is further supported by data from the German Ablation Registry which reported that despite a reduced VT burden after catheter ablation, the patients with reduced LVEF remained at an elevated risk of long-term mortality.^8^ These data suggest that better patient selection and that of procedural strategy might be necessary to improve these clinical outcomes.

Considering the overall limited representation of patients with LVEF ≤30% in previously published RCTs which have evaluated the role of VT ablation, data from real-world registries might aid in understanding clinical outcomes after VT ablation in this subset of patients. In our study, we compared outcomes after VT ablation in patients stratified by LVEF (≤30 vs. > 30%), with a primary focus on a 30-day composite safety endpoint and secondary evaluation of long-term (3 years) outcomes.

## Methods

### Study design and data source

We conducted a retrospective observational cohort study utilizing data from the TriNetX Research Network. This is a large dataset that aggregates de-identified electronic health records (EHRs) from over 115 million patients across more than 80 healthcare organizations, primarily located in the USA. The database complies with the deidentification standards specified in Section 164.514(a) of the Health Insurance Portability and Accountability Act (HIPAA) Privacy Rule. Because only aggregated de-identified data were analysed for our study, it did not require institutional review board (IRB) approval.

### Study population

Our cohort included adult patients (≥18 years) who underwent VT ablation between the dates of 1 January 2010 and 31 December 2021. Patients were identified using the Current Procedural Terminology (CPT) codes 93654. The study population was further stratified based on LVEF at the time of the ablation into two groups: those with LVEF ≤30% and those with LVEF >30%.

### Study endpoints

The primary endpoint was a 30-day composite safety outcome defined as the occurrence of any of the following: all-cause mortality, acute kidney injury (AKI), mechanical circulatory support (MCS) use, or cardiac tamponade. Individual components of the composite endpoint were analysed as exploratory outcomes. Secondary outcomes included long-term (3-year) events such as all-cause mortality, rehospitalization, and VT recurrence. Patients were followed from the date of VT ablation until the occurrence of clinical outcomes, death, or their last recorded clinical encounter in the database. For non-fatal outcomes, patients who died before the event were censored at the time of death, consistent with the Kaplan–Meier (KM) and Cox methods available in TriNetX.

Peri-procedural events were identified using structured diagnosis and procedural codes within TriNetX. For AKI and cardiac tamponade, event dates corresponded to recorded diagnosis dates in the EHR, while MCS dates were derived from procedural coding entries. These timestamps reflect documentation dates within the health system and may not represent the precise clinical onset of complications. Because all events were assessed within a fixed 30-day window after VT ablation, time-to-event analyses were performed using the recorded dates available in the database. Any imprecision in onset timing would be expected to be non-differential between matched cohorts.

### Propensity score matching

To minimize confounding, we performed 1:1 propensity score matching (PSM) using a nearest-neighbor algorithm with a caliper width of 0.1 pooled standard deviations of the logit of the propensity score. The model included demographic variables (age, gender, race/ethnicity), comorbidities (hypertension, diabetes mellitus, chronic kidney disease, chronic ischaemic heart disease, chronic obstructive pulmonary disease), and medication use (beta-blockers, antiarrhythmics, diuretics, anticoagulants, antilipaemic agents, ACE inhibitors, angiotensin II receptor blockers, calcium channel blockers, and antiplatelet agents).

### Statistical analysis

Baseline characteristics were summarized using descriptive statistics. Continuous variables were reported as means with standard deviations and compared using independent-sample Student’s *t*-tests. Categorical variables were expressed either in frequencies or in percentages and these were compared using *χ*^2^ tests. Kaplan–Meier survival analyses were performed for time-to-event outcomes, and differences between groups were evaluated using the log-rank test. Cox proportional hazards models were used to estimate hazard ratios (HRs) with 95% confidence intervals (CIs). For 30-day peri-procedural complications (AKI, MCS use, and cardiac tamponade), outcomes were primarily summarized as 30-day risks (proportions), with KM/Cox analyses presented as supportive time-to-event summaries based on recorded documentation dates. Because a single composite endpoint was prespecified as the primary outcome, no multiplicity adjustment was applied to exploratory component analyses. No formal multiplicity adjustment was applied to secondary long-term outcomes, as these analyses were prespecified as supportive and hypothesis-generating rather than confirmatory. All tests were two-tailed, and a *P*-value of <0.05 was considered statistically significant. Analyses were conducted using the TriNetX platform and R version 4.2.2, utilizing the ‘survival’ and ‘Hmisc’ packages to calculate the adjusted HRs.

## Results

We identified a total of 2549 patients who had undergone VT ablation between the dates of 2010 to 2021 and had data available on follow-up clinical outcomes. Of these, 1848 had LVEF >30% and 701 had LVEF ≤30%. Patients with LVEF ≤30% were older and had a higher incidence of co-existing clinical conditions such as chronic kidney disease, diabetes mellitus, and ischaemic heart disease. The comparison of baseline characteristics between the two patient subgroups is summarized in *Table 1*.

**Table 1:** Pre-matched characteristics

Category	Variable	EF ≤ 30 (*n* = 1848)	%	EF >30 (*n* = 701)	%	*P*-value	Std. diff
Demographics	Age at index	60.8 ± 13.5	100%	65 ± 10.6	100%	<0.0001	0.3511
	Female	557	30.26%	100	14.41%	<0.0001	0.3876
	White	1450	78.76%	534	76.95%	0.3228	0.0438
	Black or African American	207	11.24%	119	17.15%	<0.0001	0.1697
	Hispanic or Latino	58	3.15%	22	3.17%	0.98	0.0011
Diagnoses	Presence of AICD	662	35.96%	507	73.06%	<0.0001	0.8027
	Chronic ischaemic heart disease	882	47.91%	467	67.29%	<0.0001	0.4
	Primary hypertension	909	49.38%	326	46.97%	0.2808	0.0481
	Diabetes mellitus	419	22.76%	195	28.10%	0.0051	0.1228
	Chronic kidney disease	254	13.80%	232	33.43%	<0.0001	0.4751
	COPD	187	10.16%	127	18.30%	<0.0001	0.2347
Medications	Beta-blockers	1365	74.14%	562	80.98%	0.0003	0.1644
	Antiarrhythmics	1108	60.19%	535	77.09%	<0.0001	0.3706
	Diuretics	808	43.89%	499	71.90%	<0.0001	0.5917
	Anticoagulants	1005	54.59%	478	68.88%	<0.0001	0.2972
	Platelet aggregation inhibitors	944	51.28%	448	64.55%	<0.0001	0.2714
	Antilipaemic agents	926	50.30%	449	64.70%	<0.0001	0.2944
	ACE inhibitors	582	31.61%	277	39.91%	<0.0001	0.1738
	Angiotensin II inhibitors	418	22.71%	232	33.43%	<0.0001	0.2404
	Calcium channel blockers	566	30.74%	158	22.77%	<0.0001	0.1809

After 1:1 PSM, the final cohort included 623 patients in each group. Differences in baseline characteristics were minimized, with comparable (*P* > 0.05) distributions in age (64.8 ± 11.2 vs. 64.6 ± 10.6 years), race (White: 81.22 vs. 79.13%), and comorbidities such as hypertension (49.9 vs. 49.1%), diabetes mellitus (27.4 vs. 28.7%), ischaemic heart disease (66.0 vs. 66.9%), and chronic kidney disease (26.8 vs. 28.9%). Medication use was also well balanced between groups, including beta-blockers (78.9 vs. 80.4%), antiarrhythmics (75.4 vs. 75.3%), anticoagulants (67.26 vs. 66.61%), and diuretics (68.54 vs. 68.86%). Post-matched characteristics between the two patient subgroups are summarized in *Table 2*.

**Table 2:** Post-matched characteristics

Category	Variable	EF ≤ 30 (*n* = 623)	%	EF >30 (*n* = 623)	%	*P*-value	Std. diff
Demographics	Age at index	65 ± 11.1	100%	64.6 ± 10.6	100%	0.5118	0.0373
	Female	88	14.19%	96	15.48%	0.5228	0.0363
	White	488	78.71%	489	78.87%	0.9446	0.0039
	Black or African American	93	15.00%	93	15.00%	1	<0.0001
	Hispanic or Latino	19	3.07%	18	2.90%	0.8674	0.0095
Diagnoses	Presence of AICD	435	70.16%	434	70.00%	0.9505	0.0035
	Chronic ischaemic heart disease	409	65.97%	409	65.97%	1	<0.0001
	Primary hypertension	306	49.36%	303	48.87%	0.8647	0.0097
	Diabetes mellitus	179	28.87%	177	28.55%	0.9001	0.0071
	Chronic kidney disease	160	25.81%	180	29.03%	0.203	0.0724
	COPD	101	16.29%	101	16.29%	1	<0.0001
Medications	Beta-blockers	499	80.48%	495	79.84%	0.7758	0.0162
	Antiarrhythmics	464	74.84%	465	75.00%	0.9478	0.0037
	Diuretics	428	69.03%	426	68.71%	0.9024	0.007
	Anticoagulants	422	68.07%	413	66.61%	0.5858	0.031
	Platelet aggregation inhibitors	399	64.36%	392	63.23%	0.6792	0.0235
	Antilipaemic agents	394	63.55%	392	63.23%	0.9061	0.0067
	ACE inhibitors	247	39.84%	244	39.36%	0.8617	0.0099
	Angiotensin II inhibitors	202	32.58%	198	31.94%	0.808	0.0138
	Calcium channel blockers	150	24.19%	155	25.00%	0.7416	0.0187

Outcomes were analysed in two time frames: primary composite safety outcomes (peri-procedural events within 30 days) and secondary outcomes (long-term events at 3 years). For composite safety outcomes, the median follow-up was 30 days in both cohorts, with an interquartile range (IQR) of 0 days in both groups. For 3-year outcomes, the median follow-up was 1095 days in both cohorts and IQR of 402 days for the LVEF >30% group and 793 days for the LVEF ≤30% group.

### Primary outcome: 30-day composite safety endpoint

**Figure 1:**
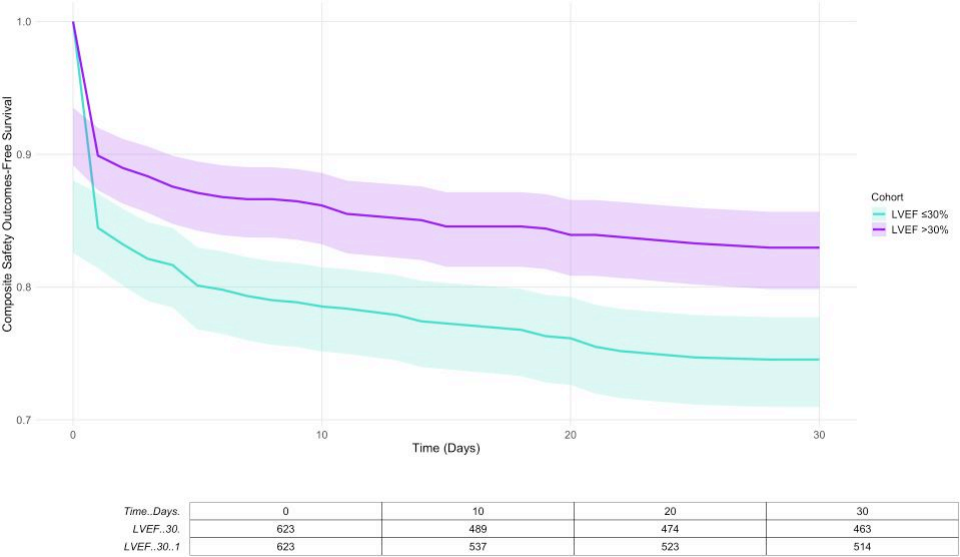
Kaplan–Meier curve for composite 30-day outcomes.

The 30-day composite endpoint (all-cause mortality, AKI, MCS use, or cardiac tamponade) occurred less frequently in patients with LVEF >30% compared with those with LVEF ≤30% (17.9 vs. 26.3%; HR 0.65, 95% CI 0.51–0.83; *P* = 0.0004). The corresponding KM curve for composite outcomes is shown in *Figure 1*.

Exploratory analyses of individual components showed that patients with LVEF >30% had lower rates of mortality (1.6 vs. 6.6%, HR 0.14, 95% CI 0.06–0.34, *P* < 0.0001), AKI (10 vs. 17%, HR 0.60, 95% CI 0.44–0.82, *P* = 0.001), and MCS use (4.0 vs. 9.5%, HR 0.42, 95% CI 0.26–0.66, *P* = 0.0001). There was no significant difference in cardiac tamponade (1.52 vs. 0.67%, HR 2.24, 95% CI 0.69–7.28, *P* = 0.47). The corresponding KM curves for individual components are shown in Supplementary material online, *Figures S1* and *S2*, *Graphical abstract*.

### Secondary outcomes: 3 years clinical outcomes

**Figure 2:**
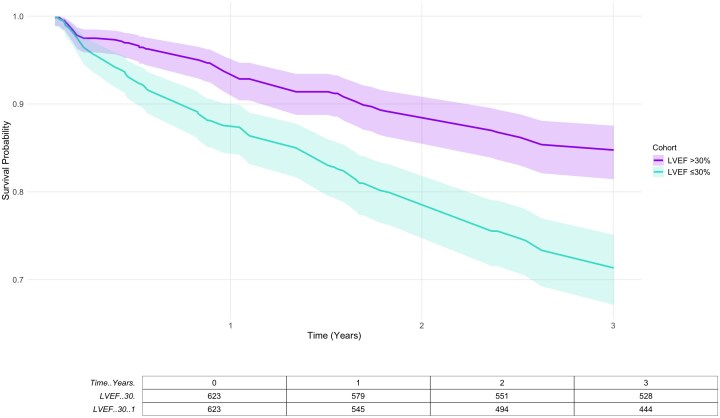
Kaplan–Meier curve for 3-year all-cause mortality following ventricular tachycardia ablation stratified by left ventricular ejection fraction.

**Figure 3:**
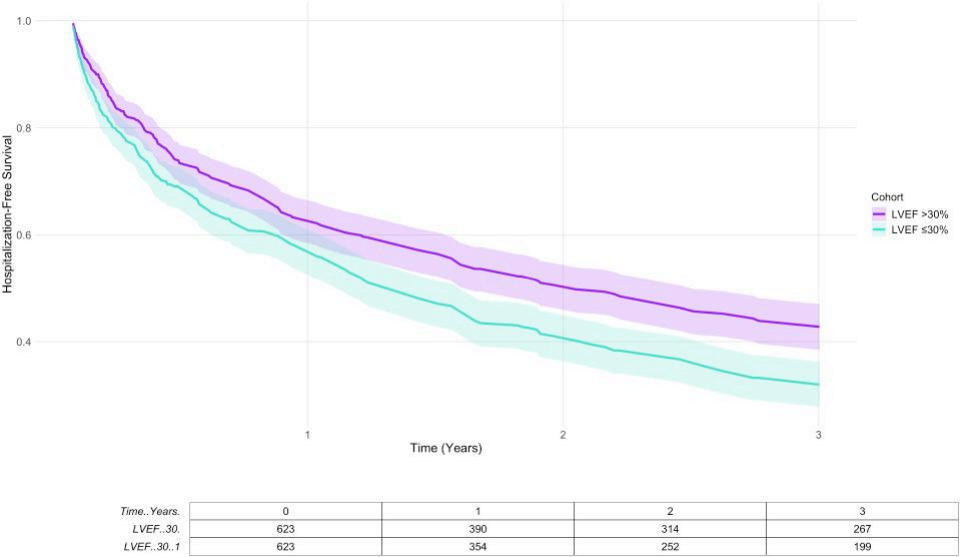
Kaplan–Meier curve for 3-year rehospitalization following ventricular tachycardia ablation stratified by left ventricular ejection fraction.

At 3 years post-ablation, all-cause mortality was significantly lower in patients with LVEF >30% compared with those with LVEF ≤30% (15.2 vs. 28.7%, HR: 0.49, 95% CI: 0.38–0.65, *P* < 0.0001). Rehospitalization rates were also lower in the higher LVEF group (31.6 vs. 44.1%, HR: 0.76, 95% CI: 0.65–0.88, *P* = 0.0003). There was no statistically significant difference in the rate of VT recurrence between the two patient subgroups (71% in patients with LVEF of > 30 vs. 67% in those with LVEF of < 30%, HR: 1.05, 95% CI: 0.91–1.20; *P* = 0.56). Kaplan–Meier curves for long-term outcomes are displayed in *Figures 2* and *3*.

## Discussion

The major findings of our study can be summarized as follows:

1. For the primary outcome, patients with LVEF >30% experienced a significantly lower rate of the 30-day composite safety endpoint compared with patients with LVEF ≤30%. Exploratory analyses of individual components demonstrated lower rates of peri-procedural mortality, AKI, and MCS use in the LVEF >30% group.
2. For secondary outcomes, patients with LVEF >30% also had more favourable 3-year long-term outcomes, including significantly lower all-cause mortality and reduced rehospitalization. However, VT recurrence remained high and did not differ significantly between groups.

In this large, real-world analysis of over 1200 propensity-matched patients undergoing VT ablation, we observed that patients with severely reduced LVEF (≤30%) experienced significantly worse outcomes compared with those with LVEF >30%. Specifically, they had higher rates of the 30-day composite endpoint, as well as increased 3-year mortality and rehospitalization, despite similar VT recurrence rates. In our study, outcomes were evaluated in two separate timeframes: primary (peri-procedural composite) and secondary (long-term), providing a comprehensive view of both short- and longer-term prognosis following VT ablation.

The findings of increased peri-procedural mortality observed in patients with LVEF ≤30% in our study are consistent with prior reports which had attributed the increase in early mortality to a greater degree of haemodynamic decompensation in this patient subset during VT ablation. In their study on 193 patients who underwent VT ablation, Santangeli *et al*.^7^ had reported a 30-day mortality of 8% in the patients who had experienced acute haemodynamic decompensation when compared with the lower risk of 3% mortality in those who did not. It is reasonable to believe that patients with a lower LVEF would be more prone to haemodynamic compromise likely due to a lower degree of contractile reserve in comparison to those with preserved or higher LVEF. While it is difficult to characterize the specific causes of mortality from the available data in TriNetX, published studies suggest that likely contributors include refractory ventricular arrhythmias, haemodynamic collapse during mapping or induction, cardiogenic shock, acute haemodynamic decompensation, and multiorgan failure.^9–11^

Patients with LVEF ≤30% also experienced significantly higher all-cause mortality during longer 3-year follow-up. This aligns with findings from 10-year follow-up data from the German Ablation Registry, which had also reported higher overall mortality in patients with heart failure with reduced ejection fraction (74.4%) compared with those with mildly reduced or preserved ejection fraction (50%).^12^ Similarly, Kumar *et al*.^13^ observed lower 6-year survival in patients with ischaemic cardiomyopathy (48%) and non-ischaemic cardiomyopathy (74%), compared with patients with no structural heart disease (100%). Collectively, these results suggest that while VT ablation may mitigate the burden of recurrent VT, it cannot alter the overall worse clinical outcomes which are seen in patients with severely reduced LV function.

In exploratory component analyses, AKI within 30 days of VT ablation was significantly higher in patients with LVEF ≤30%. This elevated risk likely reflects a combination of factors such as impaired renal perfusion, cardio-renal syndrome, underlying comorbidities, and increased susceptibility to contrast-induced nephropathy and haemodynamic instability during the procedure.^7,14^ In a multicentre study, Kuo *et al*.^15^ reported a 10% incidence of peri-procedural AKI after VT ablation and identified the presence of baseline CKD, atrial fibrillation, and haemodynamic decompensation during and after the ablation as its potential risk factors. Furthermore, AKI after VT ablation also translated to poorer survival outcomes both at early and at 1-year follow-up.^15^ Given the higher rate of AKI after VT ablation in our patient cohort of LVEF ≤30%, it is likely that this might have contributed to elevated risk of short- and long-term mortality. These findings suggest that early identification of high-risk patients and tailoring the ablation strategies accordingly to try to mitigate the risk for peri-ablation AKI. Additionally, it could also be worthwhile to implement protocols to streamline haemodynamic monitoring and utilization of MCS.

Mechanical circulatory support was more frequently employed in patients with LVEF ≤30%, reflecting the increased procedural risk and haemodynamic instability inherent to this group. While direct comparisons of MCS usage in this high-risk population are limited, our findings align with prior data from the National Inpatient Sample, which had reported a utilization rate of 6.5% in patients who underwent VT ablation.^16^ Similarly, in a large cohort of 41 075 VT ablation hospitalizations, short-term MCS was used in 6.0% of cases, with a trend of increased utilization from 4.3% in 2010 to 6.8% in 2017.^17^ However, despite the increasing trend; the overall benefit of use of MCS both in terms of its timing and which patient subgroup benefit the most from its use have not been fully defined. Although some observational studies suggest the use of MCS might facilitate activation mapping of haemodynamically unstable VT; a recent meta-analysis found no significant difference in VT recurrence or mortality between patients who received pre-emptive MCS and those who did not.^18^ Although recent studies have supported the utilization of substrate modification as well as targeting functional substrate in sinus or in paced rhythm as an obviating strategy for haemodynamic instability, there is not enough evidence for wider clinical adoption.^19^

The incidence of cardiac tamponade was overall low and did not differ between the LVEF >30% and LVEF ≤30% groups (1.52 vs. 0.67%). In large VT ablation studies, cardiac tamponade was reported in ∼1.0–1.5% of procedures.^20,21^ The observed event rates in our cohort are consistent with these prior reports. Although we included a substantial proportion of patients with severely reduced LVEF, the overall incidence of tamponade continued to be relatively low. Previously published studies^20,21^ had not reported the risk of cardiac tamponade according to the baseline LV function or comorbidity burden. Nevertheless, our findings align with broader trends showing that, while pericardial complications remain a serious concern, their absolute rates are low.

In our study, patients with LVEF ≤30% had significantly higher rates of hospitalizations at 3-year follow-up in comparison to the patient subgroup with LVEF >30%. This increased burden likely reflects not only the recurrence of VT, but also progressive heart failure, and co-existing comorbidities. Lee *et al*.^9^ reported that patients with persistent heart failure continued to experience higher rates of readmissions and worsening of functional status despite having had successful VT suppression after ablation. These findings also suggest that despite a mitigated ventricular arrhythmia burden, those with advanced heart failure remained at an elevated risk of their overall cardiac status.

Ventricular tachycardia recurrence within 3 years occurred in 71.0% of patients with LVEF >30% and 67.0% of those with LVEF ≤30%, with no statistically significant difference between groups. These high recurrence rates reflect the ongoing challenges of arrhythmia suppression in patients with structural heart disease. Kumar *et al*.^13^ reported ventricular arrhythmia recurrence rates of 46% in ischaemic and 62% in non-ischaemic cardiomyopathy over 6 years, while Aldhoon *et al*.^22^ reported a recurrence in 67% of patients who had undergone VT ablation for electrical storm and a recurrence rate of 60% in those without pre-ablation electrical storm over a follow-up period of 2.5 years.^13,22^ Moreover, the VTACH trial reported that 53% of patients experienced VT recurrence within 2 years of having undergone VT ablation.^4^ A recent analysis also demonstrated higher 1-year VT recurrence rates in patients with lower LVEF, with recurrence occurring in 36.7% of those with LVEF <25% and 32.3% of those with LVEF 25–49%.^23^ The lack of significant difference in post-ablation recurrence rate of VT in our study may reflect the high baseline arrhythmic burden in both cohorts and the limitations of current ablation strategies in altering long-term substrate progression. Importantly, these findings do not suggest that ablation was ineffective in either group. Prior studies, including VANISH,^5^ have shown that even when complete arrhythmia suppression is not achieved, ablation can reduce the burden of VT and along with lowering the risk of electrical storm and ICD shocks. The persistent divergence in mortality and rehospitalization despite similar recurrence rates suggests that ablation offers clinical benefit, but underlying myocardial dysfunction may be the dominant driver of outcomes.

Several pathophysiologic mechanisms likely contribute to the poorer outcomes observed in patients with low LVEF. These mechanisms might involve the presence of extensive myocardial scarring and relatively larger arrhythmia substrate which might not only limit the effectiveness of ablation but might also contribute to poorer contractile reserve in achieving durable rhythm control.^24^ Chronic neurohormonal activation, through heightened sympathetic drive and renin–angiotensin–aldosterone system stimulation, promotes adverse remodelling and increases the risk of both arrhythmias and pump failure.^25^ These adverse pathophysiological and maladaptive changes might not be completely ameliorated by the VT ablation as a standalone measure. Autonomic imbalance, including reduced vagal tone and increased sympathetic activity, further exacerbates electrical instability and impairs haemodynamic responses during VT.^25^ Additionally, renal dysfunction is common in this population due to low cardiac output and systemic congestion, contributing to cardio-renal syndrome and further clinical deterioration.^26^ While ablation can modify arrhythmogenic substrate, it does not address these systemic processes. These findings highlight the need for integrated care strategies that combine ablation with early evaluation for advanced heart failure therapies to improve outcomes in this high-risk group.

Our study represents a relatively large real-world analysis of outcomes after VT ablation in patients stratified by LVEF. The use of a large, multi-institutional database, aggregating data from over 115 million patients across diverse healthcare organizations, enhances the generalizability and external validity of our findings to real-world clinical practice. We utilized a rigorous 1:1 PSM to balance key demographic, clinical, and medication variables between the LVEF ≤30% and LVEF >30% groups, which helped to mitigate confounding and supported a more accurate comparative analysis. The extended follow-up period of up to 3 years also strengthens the ability to assess both peri-procedural and long-term outcomes, providing valuable insights into the real-world prognosis of patients undergoing VT ablation across different degrees of ventricular dysfunction.

### Limitations

We acknowledge several important limitations of our study. Ours is an observational study and despite rigorous PSM, residual confounding due to unmeasured variables cannot be fully excluded. Moreover, misclassification of clinical outcomes is also possible because of reliance on the administrative coding within EHR data, although some of this risk is mitigated using standardized procedural and diagnostic codes and any misclassification is likely to be balanced between the two cohorts we evaluated. In addition, peri-procedural complications were identified using recorded diagnosis and procedure dates in TriNetX, which may reflect documentation or discharge dates rather than the precise clinical onset of events. Therefore, temporal imprecision in the exact timing of AKI, MCS use, and cardiac tamponade relative to the index procedure cannot be fully excluded. We also did not have available data on the specific causes of mortality in the TriNetX database, which limited our ability to determine whether deaths were attributable to arrhythmic events, heart failure progression, or non-cardiac causes. Additionally, we also did not have information on the procedure-specific characteristics (e.g. substrate modification strategies, activation mapping techniques, and the use of epicardial access), patient selection criteria, decisions regarding prophylactic MCS use, and rates of repeat ablation procedures. We were also unable to determine whether VT ablations were performed electively or in the setting of acute clinical instability (e.g. electrical storm or inpatient decompensation), as procedural acuity and indication are not reliably captured in structured TriNetX data fields. Procedural acuity may influence both baseline risk and subsequent outcomes and therefore represents a potential source of residual confounding. Because deaths prior to non-fatal outcomes were treated as censoring events rather than terminal competing events, our analysis may overestimate the cumulative incidence of certain non-fatal outcomes. We acknowledge that this is an important limitation of the TriNetX platform, which does not support competing-risks models.

## Conclusions

In this large, real-world analysis of patients undergoing VT ablation, severely reduced LVEF (≤30%) was associated with significantly higher peri-procedural complications, long-term mortality, and rehospitalization compared with patients with relatively preserved LV function. These findings underscore the need for careful risk stratification, peri-procedural support strategies, and early consideration of advanced heart failure therapies in those with advanced ventricular dysfunction undergoing VT ablation. Future studies are warranted to optimize procedural approaches and long-term management strategies for this high-risk population.

## Lead author biography


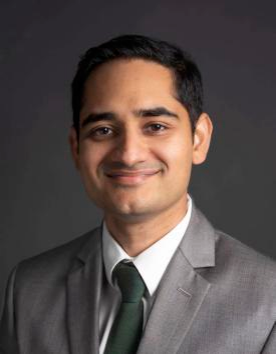


Dharmindra Dulal, MD is an Internal Medicine resident at The Ohio State University Wexner Medical Center with a strong academic focus on cardiovascular medicine and cardiac electrophysiology. He earned his medical degree from University of Toledo College of Medicine and Life Sciences and holds a Bachelor of Science in Biology and Chemistry from Radford University. Dr Dulal has developed a robust research portfolio centred in arrhythmia management, particularly catheter ablation in complex and high-risk populations. His research interests include atrial fibrillation, ventricular tachycardia, cardio-oncology, and the intersection of electrophysiology with systemic disease. He has authored multiple peer-reviewed publications, including first-author studies in journals such as European Heart Journal—Quality of Care and Clinical Outcomes and Heart Rhythm, and has presented his work at multiple national conferences. His recent work on ventricular tachycardia ablation outcomes stratified by left ventricular ejection fraction highlights his commitment to clinically impactful, methodologically rigorous research. Beyond research, Dr Dulal is deeply engaged in advancing evidence-based cardiovascular care and aims to pursue fellowship training in cardiology with a focus on electrophysiology. His academic approach emphasizes careful study design, critical appraisal of data, and translating real-world evidence into meaningful clinical insights.

## Supplementary Material

oeag057_Supplementary_Data

## Data Availability

The data that support the findings of this study are available from TriNetX, LLC but third-party restrictions apply to the availability of these data. The data were used under license for this study with restrictions that do not allow for the data to be redistributed or made publicly available. However, for accredited researchers, the TriNetX data are available for licensing at TriNetX, LLC. Data access may require a data sharing agreement and may incur data access fees.
