# Supplementary figures and images for: Impact of left ventricular ejection fraction on clinical outcomes following ventricular tachycardia ablation: a propensity-matched analysis from a large multicentre database
Source: Eur Heart J Open. 2026 Apr 9;6(2):oeag057. doi: 10.1093/ehjopen/oeag057 (PMC13127762; doi:10.1093/ehjopen/oeag057)

# Study Methodology: VT Ablation Outcomes by LVEF Stratification

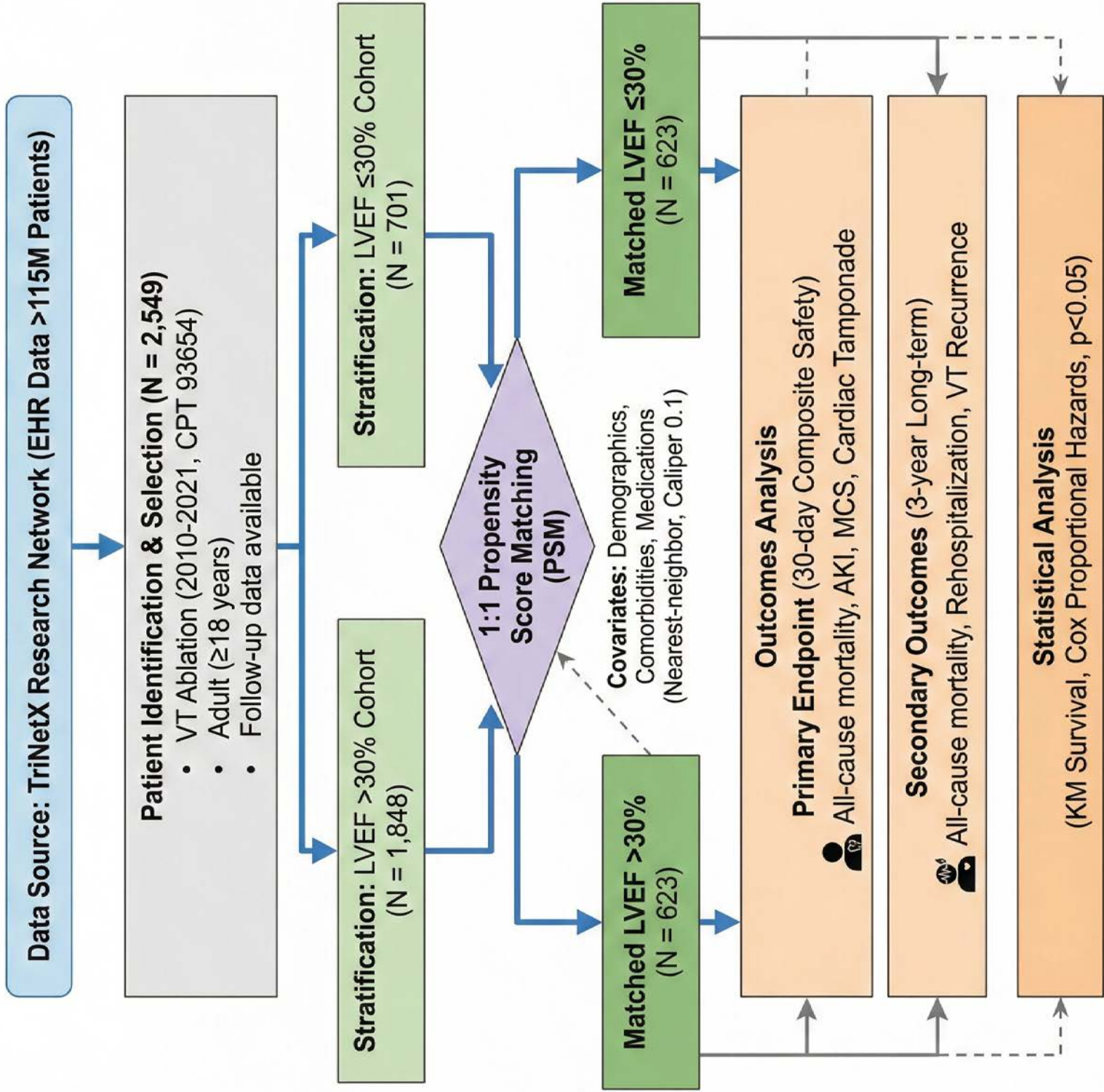

Supplement: oeag057_Supplementary_Data [file oeag057_supplementary_data.zip › LVEF_Flowchart.pdf]

Forest Plot: Outcomes After VT Ablation by LVEF (>30% vs ≤30%)

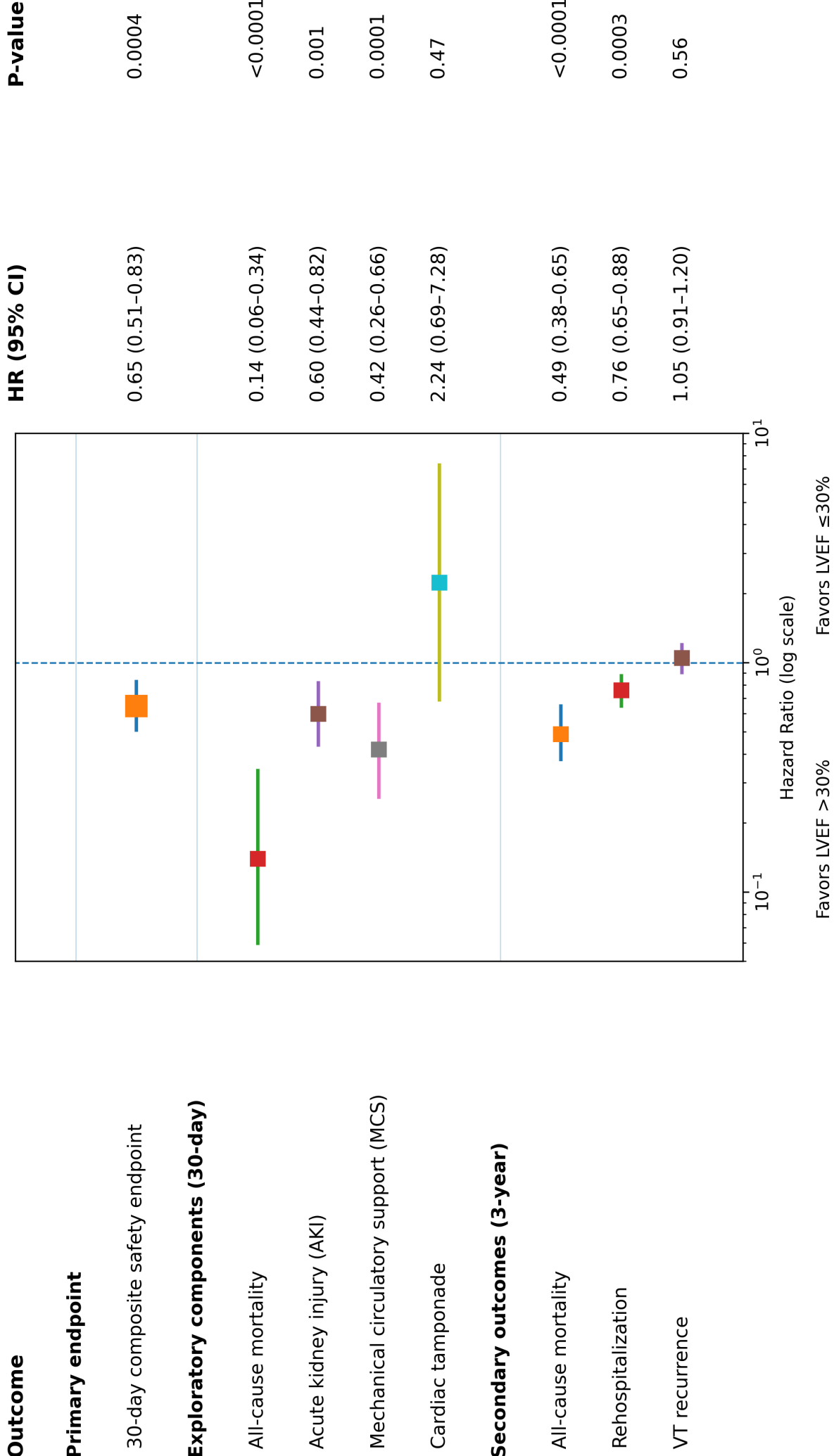

Supplement: oeag057_Supplementary_Data [file oeag057_supplementary_data.zip › VT_Forest_Plot.pdf]
